# MiR-429 Involves in the Pathogenesis of Colorectal Cancer via Directly Targeting LATS2

**DOI:** 10.1155/2020/5316276

**Published:** 2020-12-19

**Authors:** Xia Chen, Ai-li Wang, Yuan-yuan Liu, Chen-xi Zhao, Xue Zhou, Hai-long Liu, Mo-bin Lin

**Affiliations:** ^1^Center for Clinical Research and Translational Medicine, Yangpu Hospital, Tongji University School of Medicine, Shanghai 200090, China; ^2^Institute of Gastrointestinal Surgery and Translational Medicine, Tongji University School of Medicine, Shanghai 200090, China; ^3^Department of General Surgery, Yangpu Hospital, Tongji University School of Medicine, Shanghai 200090, China

## Abstract

Colorectal cancer (CRC) is a leading cause of cancer-related death around the world whose recurrence and metastasis rate is high. Due to the underlying unclear pathogenesis, it is hard so far to predict the tumorigenesis and prevent its recurrence. YAP/TAZ has been reported to be activated and functioned as a potential oncogene in multiple cancer types and proved to be essential for the carcinogenesis of most solid tumors. In the present study, we found that YAP/TAZ was markedly upregulated in CRC tissues comparing with the adjacent noncancerous tissues due to the downregulation of LATS2, the main upstream regulator. We further identified miR-429 as a direct regulator of LATS2-YAP/TAZ activation, suggesting that the miR-429-LATS2-YAP/TAZ might be novel effective diagnostic axis and therapeutic targets for CRC.

## 1. Introduction

Colorectal cancer (CRC) is one of the most leading causes of cancer-related deaths around the world [1]. It was estimated to have 1.4 million new cases and about 0.7 million global deaths in 2018 [2]. It took over 9.2% of cancer-related mortality. The survival rate for colorectal cancer patients is 12 to 18 months. The five-year survival rate is 65% [3]. Conventional surgery and/or chemotherapy are usually the most frequently used treatments. Despite the advances in diagnosis and treatment, CRC patients often relapse and metastasize [4]. Genetic alterations such as activation of proto-oncogenes or loss of function of tumor suppressor genes are the main pathogenesis of CRC. Nevertheless, because of the knowledge shortage about the molecular basis of CRC, there is up to now no effective method to predict oncogenesis and prevent its recurrence. It is extremely important to identify more novel effective diagnostic biomarkers and therapeutic methods for CRC. The purpose of this study is to explore key genes as potential biomarkers for CRC diagnosis and prognosis for clinical utility.

Multiple signaling pathways are dysregulated due to genetic or epigenetic alterations in cancer. Hippo signaling, an evolutionarily conserved signaling pathway for tissue growth, has been proven to be a significant regulator in tumor biology by inducing target gene transcription and enabling downstream biological effects [5, 6]. As the key downstream mediator of the Hippo signaling pathway, YAP/TAZ (Yes-associated protein and its transcriptional coactivator with PDZ-binding motif) have been reported to be activated and functions as potential oncogenes in multiple cancer types such as oral squamous cell carcinoma [7], glioma [8, 9], bladder cancer [10], liver cancer, breast cancer, and lung cancer. Furthermore, the protein levels and activities of YAP/TAZ in cancer stem cells of breast and osteosarcomas were elevated and were indispensable to maintain the self-renewal and tumor-initiation abilities [11–13]. These results suggested that YAP/TAZ might play important roles in the development of most tumors or cancers.

Roles and regulations of Hippo/YAP signaling were extensively studied in cancer biology. Studies showed that overexpression of YAP/TAZ could reduce the survival time in CRC [14] patients and was found to mediate the tumor suppression effect of the medicine JieduSangen Decoction in colon cancer [15]. Overexpression of YAP attenuated the inhibitory effects of the lncRNA B4GALT1-AS1 in colon cancer [16]. YAP was also found to mediate tumor survival enabled by Cucurbitacin B [17, 18] and GPRC5A [19]. However, the exact expression profiles and features of YAP/TAZ in colon cancer have not been elucidated yet.

microRNAs are a class of evolutionarily conserved noncoding small RNAs that usually regulate gene expression at the posttranscriptional level. They may regulate as many as 30 percent of human genes by controlling the stability and translation of target mRNAs and then modulate various cellular processes such as proliferation, differentiation, apoptosis, and angiogenesis [20, 21]. It is believed that one mRNA could be regulated by diverse microRNAs, and one microRNA may target various mRNAs. The diversity and regulation functions of microRNAs have been uncovered in many diseases including cancers in recent years.

The current study aims to find out the biological roles and mechanisms of YAP/TAZ expression in colon cancer and shed a light on the development and prognosis of CRC. We characterized YAP/TAZ expressions in colon adenocarcinoma (COAD) and identified a new regulator of their expressions by finding out that miR-429 modulated YAP/TAZ expression via targeting LATS2. We also assessed the impacts of miR-429 on CRC cell growth both in vitro and in vivo. We demonstrated that YAP/TAZ signaling regulated by miR-429 promoted cell proliferation and tumor growth of COAD through targeting LATS2.

## 2. Materials and Methods

### 2.1. Human Clinical Samples

In the present study, 21 pairs of human COAD tissues and corresponding nontumor control tissues were obtained from Yangpu Hospital, Tongji University. Informed consent of all CRC patients was acquired before the experiment. The study was approved by the Institutional Ethics Committee of Tongji University.

The RKO and HCT116 CRC cell lines were obtained from ATCC. All cells were cultured in Dulbecco's modified Eagle's medium (DMEM) supplemented with 10% fetal bovine serum (FBS, Gibco), 1% penicillin, and 1% streptomycin (Life Technologies).

### 2.2. Immunohistochemistry

The slides were dewaxed in xylene twice for 10 min, rehydrated in successive concentrations of ethanol (100%, 95%, and 80%) and distilled water for 5 min each, and heated in 0.01 M, pH 6.0 sodium citrate buffer for antigen retrieval. Endogenous peroxidase was then eliminated with fleshly prepared 3% hydrogen peroxide for 20 min. Nonspecific staining was blocked by incubation in 5% BSA for 2 hrs. The slides were then blotted with a 1 : 100 diluted YAP antibody (CST, Danvers, USA) or TAZ antibody (CST, Danvers, USA) at 4°C overnight, followed by incubation with HRP conjugated secondary antibody (Abmart, Shanghai, China) for 2 hrs at room temperature. Immunostaining was performed with the DAB Horseradish Peroxidase Color Development Kit (Beyotime, Shanghai, China), and counterstain was performed with hematoxylin. Positive reactions were defined as those showing brown signals. The expression levels of YAP/TAZ were evaluated by the IHC score, which was calculated by multiplying a proportion to 2, ≥25%; 3, >25 to 50%; 4, >50%, and the intensity score revealed the staining intensity (no staining, score 0; weak, score 1-3; intermediate, score 4-6; strong, score greater than 6).

### 2.3. Short Hairpin RNA Interference

To stably knockdown the expression of miR-429, we designed 7 shRNA sequences that directly target the stem-loop structure of pre-miRNA-429. The related oligos were chemically synthesized by Guangzhou RiboBio Co. Ltd (Guangzhou, China). Oligos were resolved into ddH_2_O at a concentration of 100 *μ*M and annealed using Annealing Buffer for DNA Oligos (D0251, Beyotime Biotechnology, Shanghai, China) by a program of naturally cooling from 100 to 25°C. Annealing products were diluted (1/100) using ddH_2_O and subjected to ligation into pLKO.1 vector (#10878, Addgene, Watertown, USA). Only three shRNAs that efficiently knockdown miR-429 were listed as follows. sh-miR-429_#1: Forward, 5′-CCG GCC GGC CGA TGG GCG TCT TAC CTC GAG GTA AGA CGC CCA TCG GCC GGT TTT TC-3′; Reverse, 5′-AAT TGA AAA ACC GGC CGA TGG GCG TCT TAC CTC GAG GTA AGA CGC CCA TCG GCC GG-3′; sh-miR-429_#2: Forward, 5′-CCG GGC CGA TGG GCG TCT TAC CAG CTC GAG CTG GTA AGA CGC CCA TCG GCT TTT TC-3′; Reverse, 5′-AAT TGA AAA AGC CGA TGG GCG TCT TAC CAG CTC GAG CTG GTA AGA CGC CCA TCG GC-3′; sh-miR-429_#3: Forward, 5′-CCG GTT AGA CCT GGC CCT CTG TCT CTC GAG AGA CAG AGG GCC AGG TCT AAT TTT TC-3′; Reverse, 5′-AAT TGA AAA ATT AGA CCT GGC CCT CTG TCT CTC GAG AGA CAG AGG GCC AGG TCT AA-3′.

Plasmids were then transfected into 293 T cells with the packaging plasmid psPAX2 and the envelope plasmid pMD2.G using FuGENE® 6 transfection reagent (Roche Applied Biosciences, Switzerland) to package the lentivirus. After 24 hrs and 48 hrs of transfection, the cell culture media were collected and mixed, centrifuged at 1300 rpm for 5 min, and then filtered through a 0.45-*μ*m filter and aliquoted. The viral supernatants supplemented with 8 *μ*g/ml polybrene (Sigma-Aldrich) were used to infect target cells. 24 hrs later, the infected cell media were changed to fresh medium supplemented with 2.5 *μ*g/ml puromycin (Sigma-Aldrich) every two days for one week.

### 2.4. Quantitative PCR Amplification

Total RNA was extracted and further reversely transcribed into cDNA by using a PrimeScript™ II 1st Strand cDNA Synthesis Kit (Takara, Japan). Gene expression was detected by real-time quantitative PCR amplification with SYBR Premix Ex Taq™ (Tli RNaseH Plus) (Takara, Japan) mixed with cDNA templates and forward and reverse primers. Real-time PCR reactions were conducted on an ABI 7500 Real-Time PCR System with a two-step PCR procedure (denaturing at 95°C for 30 sec followed by 40 cycles of 95°C, 5 sec and 65°C, 30 sec). *β*-Actin was used as an endogenous loading reference. The *ΔΔ*Ct quantification method was used to analyze the relative mRNA expression levels of genes. The oligos involved in the study were listed as follows: YAP: Forward, 5′-CCC CCA CTG GAG TAG TCT CT-3′, Reverse, 5′-GAC AGC ATG GCC TTC CTG GG-3′; TAZ: Forward, 5′-CCG TCA GTT CCA CAC CAG TG-3′, Reverse, 5′-TGG ATT CTC TGA AGC CGC AG-3′; LATS1: Forward, 5′-CCC TCA AGC TGT CGA TGT GG-3′, Reverse, 5′-CTT GCC TAA GCG ATC TTC GG-3′; LATS2: Forward, 5′-GGG CTT CAT CCA CCG AGA CA-3′, Reverse, 5′-GCC CTC TGC TCT AGG GTC TT-3′.

For microRNA analysis, RNA was converted to cDNA using the miScript II RT Kit (Qiagen, USA), and real-time PCR was performed using a miScript Primer Assay (forward primer) and the miScript SYBR Green PCR Kit in a final volume of 10 *μ*L (Qiagen, USA) and normalized on RNU48 and RNU49 endogenous controls.

### 2.5. Western Blot Analysis

Tissues/cells were lysed using RIPA lysis buffer that was freshly added protease inhibitor cocktail (Roche, Switzerland) to extract total proteins. Protein concentration was determined by Pierce™ BCA Protein Assay Kit (ThermoFisher, USA). 20 *μ*g protein was used for each lane to electrophoresis in SDS-PAGE gel and transferred onto a 0.22-*μ*m PVDF (polyvinylidene difluoride) membrane (ThermoFisher, USA). After transferred, the membranes were immersed in 5% nonfat milk for 1 h at room temperature to block nonspecific reaction and incubated with the primary antibodies of LATS2 (Abcam, USA), YAP (Abcam, USA), TAZ (Abcam, USA), and GAPDH (Invitrogen, USA) overnight at 4°C. Horseradish peroxidase-conjugated secondary mouse anti-rabbit or goat anti-mouse antibodies (Cell Signaling Technologies, USA) were blotted for 1 hr at room temperature. Finally, Tanon™ High-sig ECL Western Blotting Substrate (Tanon, Shanghai, China) was used for the detection of protein expression levels.

### 2.6. Colony Formation Assay

3000 cells were seeded onto each 6-well plates well and cultured for 2 weeks. The cells were fixed in 100% methanol for 20 min and then stained with 0.25% crystal violet solution and analyzed. Colonies were determined under a microscope. A mass with ≥50 cells was recognized as a colony. The efficiency of colony formation was figured out as (number of colonies/number of cells inoculated) × 100%.

### 2.7. Luciferase Reporter Assay

The wild type and mutant 3′ UTR sequence of LATS2, respectively, LATS2-WT and LATS2 3′-MUT, were amplified using PCR amplification assay and cloned into the luciferase reporter vector pGL3-Basic (Promega, USA) to construct the pGL3-LAST2 and pGL3-LAST2-mut plasmids. Target cells were seeded onto 96-well plates and pGL3-LAST2 or pGL3-LAST2-mut plasmid transfected together with the Renilla reporter plasmid pRL-TK using the Lipofectamine 3000. In the meantime, either a miR-429 mimic or negative control was also cotransfected. After 48 hrs of transfection, cells were lysed with PLB, and the activities of the Firefly and Renilla luciferase were successively examined using the luciferase assay reagent II and stop&glo reagent of the Dual-Luciferase Reporter Assay System (Promega, USA).

### 2.8. In Vivo Animal Studies

Animal studies were conducted following the National Academy of Sciences rules for the care and use of laboratory animals. Animal experiments were approved by the laboratory animal care committee of Tongji University. Female BALB/c athymic nude mice of 4-6 weeks old and 16-20 g weight were purchased from Shanghai SLAC Laboratory Animal Co. Ltd.

To establish a tumor xenograft model, the nude mice were randomly divided into four groups, sh-miR-429-1, sh-miR-429-2, sh-miR-429-3 groups, and control group. Each group had 3 mice, and tumor xenografts were made by bilateral axillary injection. Briefly, the mice were anesthetized with 2% pentobarbital sodium at a dose of 40-45 mg/kg body weight, and HCT116/RKO cells carrying sh-miR-429 or mock cells diluted in 100 *μ*L PBS were subcutaneously injected into the mice (3 × 10^6^/mouse). Tumor sizes were measured with a caliper every 5 days, and the tumor volumes were calculated as follows: tumor volume (mm^3^) = 0.5 × length × width^2^. Tumor weights were weighed out using an analytical balance.

### 2.9. Statistical Analysis

Data were expressed as mean ± SD from at least three independent experiments. The significance of differences was determined with Student's *t*-test or ANOVA test. *P* values were calculated, and *P* < 0.05 was considered as statistically significant. Asterisks were assigned as follows: ^∗^*P* < 0.05, ^∗∗^*P* < 0.01, ^∗∗∗^*P* < 0.001, and n.s.: no significance.

## 3. Results

### 3.1. YAP/TAZ Is Activated in COAD Tissues

**Figure 1:**
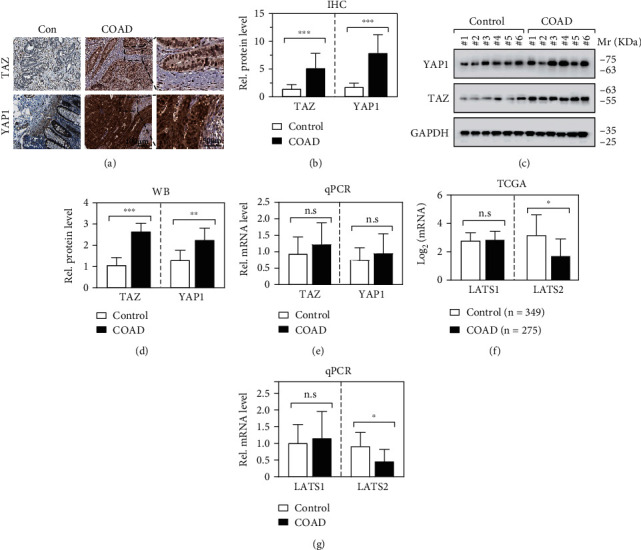
YAP/TAZ is activated in COAD. (a, b) Representative examples of immunostaining images and column diagrams for YAP1 and TAZ in the 21 COAD tumors and corresponding noncancerous tissues. (c, d) Western blotting analysis of YAP/TAZ protein expression in the paired COAD tissues. (e) mRNA expression levels of YAP1 and TAZ in COAD. (f) Bioinformatic analysis in TCGA for LATS1 and LATS2 expressions. (g) Quantitative PCR amplification verification of LATS1 and LATS2 expression in the collected 21 paired COAD tissues.

To explore the clinical role of YAP/TAZ in colon cancer, we first examined their expression levels in the paired colon cancer and noncancerous tissues by immunohistochemistry staining. Results showed that the YAP1 and TAZ expressions were significantly upregulated in the COAD tissues (Figures 1(a) and 1(b)). Western blotting hybridization further confirmed that the protein levels of YAP1 and TAZ markedly increased in the 21 paired COAD tissues compared to the surrounding noncancerous tissues (Figure 1(c) and 1(d)). However, it was interesting for us to find out that there were no statistically significant differences of YAP1 and TAZ between COAD and the control at the mRNA levels (Figure 1(e)), suggesting that the different expression levels might occur at the posttranscriptional stage.

### 3.2. LATS2 Is Related to YAP/TAZ Activation in COAD

It is well known that activated LATS1/2 suppresses the activities of YAP/TAZ by directly phosphorylate multiple serine residues in YAP/TAZ [22–24]. To investigate whether it was LATS1/2 kinases that affected YAP/TAZ protein levels in COAD, we first analyzed the colon cancer TCGA dataset with 275 COAD and 349 control tissues. Notably, the expression level of LATS2 but not LATS1 was remarkably downregulated in COADs compared to normal controls (Figure 1(f)). Quantitative PCR amplification in the 21 paired COAD tissues further verified that the LATS2 expression was downregulated (Figure 1(g)).

### 3.3. miR-429 Is Associated with LATS2 in COAD Samples

**Figure 2:**
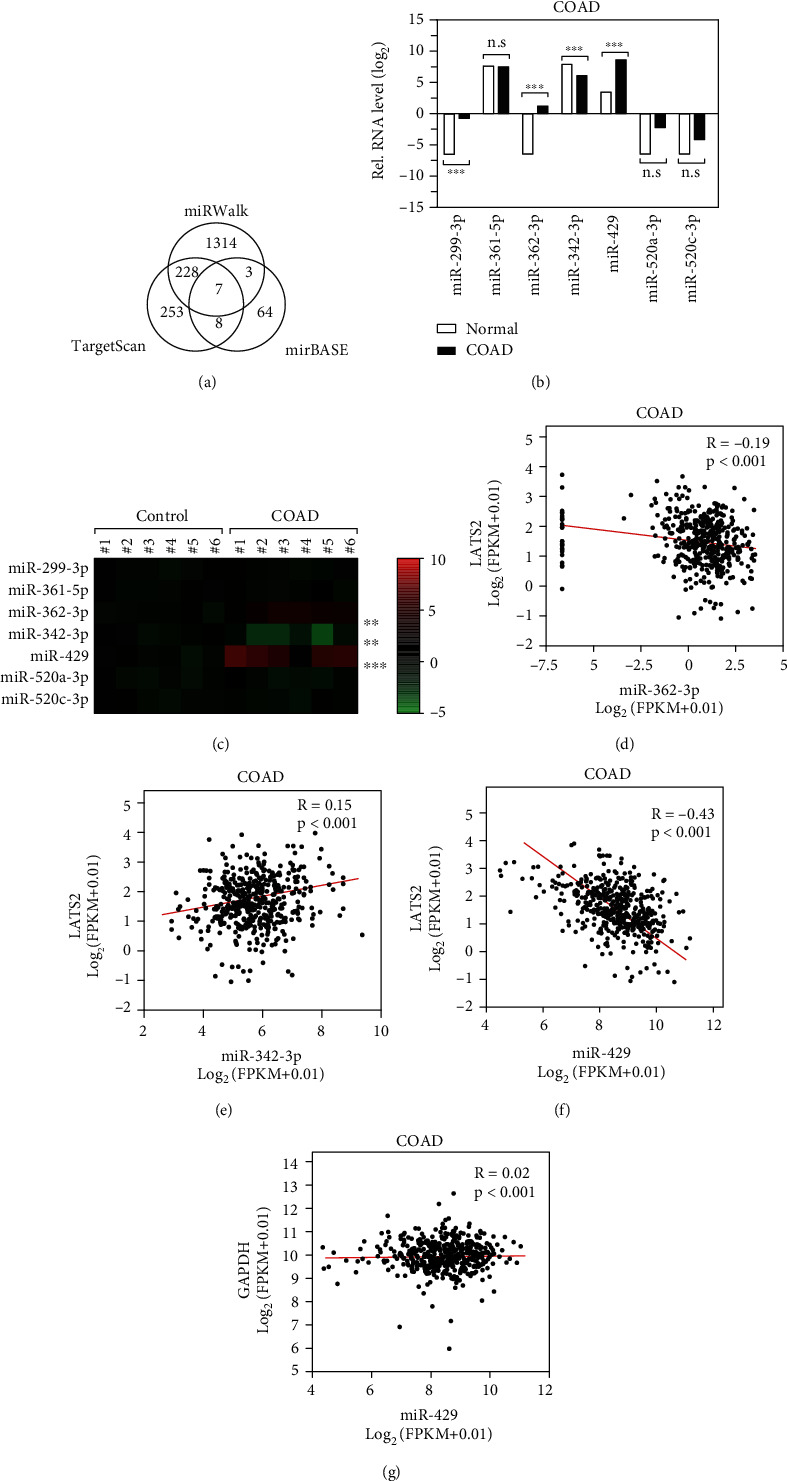
miR-429 is associated with LATS2 in COAD samples. (a) Databases searching of potential microRNAs for LATS2. (b) Expression profiles of the microRNAs in the ENCORI Pan-Cancer Analysis Platform. (c) Heatmap of the microRNAs expression in the 6 representative paired COAD tissues. (d–g) Statistical of the correlation of LATS2 and the representative miRNAs.

microRNAs (miRNAs) have been reported to be involved in various pathological processes including cancer. The rapid development of bioinformatic technology provides a convenient way for the identification of miRNA target genes. After we analyzed the most common three miRNA databases including TargetScan, miRWalk, and miRBase for the putative miRNA regulators of LATS2, we found a total of 7 joint miRNAs dysregulated, which were miR-361-5p, miR-299-3p, miR-362-3p, miR-342-3p, miR-429, miR-520a-3p, and miR-520c-3p (Figure 2(a)). Data from the TCGA colorectal cancer miRNA sequencing dataset indicated that the expression levels of miR-429, miR-299-3p, and miR-362-3p were significantly upregulated, while the level of miR-342-3p was downregulated in the COAD tissues compared to normal (Figure 2(b)). Then, we detected the expression patterns of these miRNAs in the paired colon cancer tissues and noted that the expression levels of miR-429 were most significantly higher in the COAD tissues as shown in the heat map (Figure 2(c)). To give insight into whether these miRNAs were regulators of LATS2, we investigated the relationship between the levels of these miRNAs and LATS2 expression in the database. As expected, data analysis showed that the miR-429 level was highly inversely correlated with LATS2 (*R*^2^ = −0.43, *P* < 0.001), suggesting that miR-429 was a noteworthy miRNA that downregulates LATS2 in CRC (Figure 2(d)–2(g)).

### 3.4. sh-miR-429 Inhibits Tumor Cell Growth In Vitro

**Figure 3:**
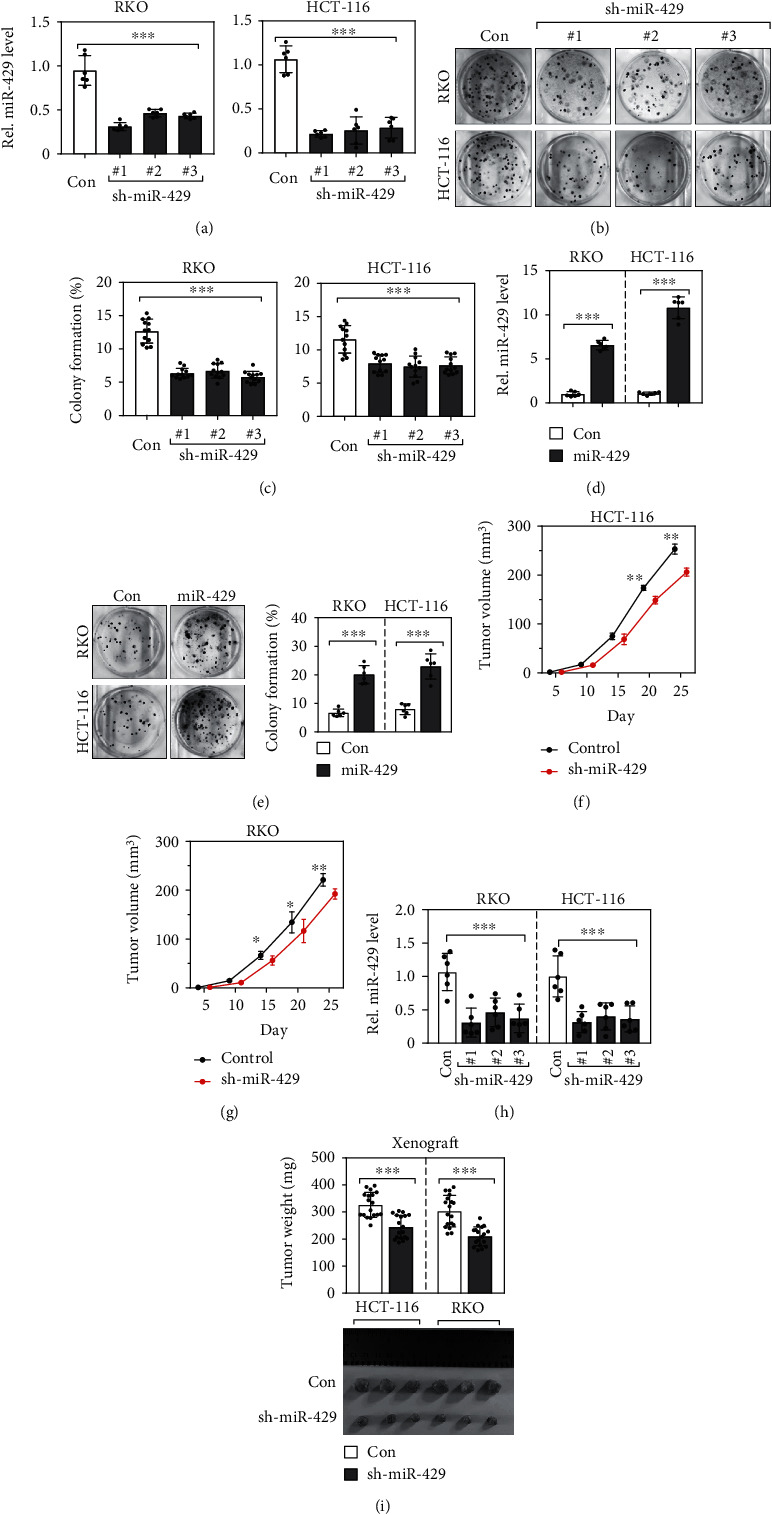
Sh-miR-429 inhibits tumorigenesis both in vitro and in vivo. (a) Knockdown of miR-429 in RKO and HCT116 cell lines by short hairpin RNA technology. (b, c) Knockdown of miR-429 reduced the colony formation ability of RKO and HCT116 cells. (d) Forced expressions of miR429 in RKO and HCT116 cell lines. (e) Overexpression of miR429 increased the colony formation ability of RKO and HCT116 cells. (f, g) sh-miR-429 reduced tumor growth rates of RKO and HCT116 xenografts in nude mice (*n* = 6/group). (h) shRNA interfering knock down the expression levels of miR-429 in the xenografts. (i) sh-miR-429 reduced tumor weights and dimensions of RKO and HCT116 xenografts in nude mice (*n* = 6/group).

To determine whether miR-429 confers tumor-progressive function in CRC cells, miR-429 was knocked down via shRNA in RKO and HCT116 cells by stably infecting RKO and HCT116 cells with lentiviral constructs carrying one of three miR-429-based shRNAs or with a scrambling vector as control. Quantitative RT-PCR analysis revealed a marked reduction of miR-429 levels in RKO and HCT116 cells after knocking down (Figure 3(a)). The colony formation abilities of RKO and HCT116 cells were significantly reduced following knockdown of miR-429 (Figures 3(b) and 3(c)). On the contrary, forced expression of the miR-429 mimics yielded the opposite effects, which greatly increased the colony-forming ability than the corresponding control (Figure 3(e)).

### 3.5. sh-miR-429 Inhibits Tumorigenesis in vivo

Given the abovementioned results, we hypothesized that the weakened expression of miR-429 might suppress CRC progression and development. Therefore, we further examined the effect of miR-429 on CRC tumor progression in vivo. The resulting RKO and HCT116 cells carrying sh-miR-429s or control were subcutaneously injected into the athymic nude mice to create a subcutaneous xenograft model. Tumor volumes were evaluated every day. Tumor weights were measured when the mice were sacrificed four weeks later. In line with our in vitro findings, the results showed that miR-429 depletion exhibited significantly slower growth than those with the control as evidenced by reduced tumor weights (Figures 3(h) and 3(i)) and tumor volumes (Figures 3(f) and 3(g)), implying that miR-429 is actually involved in CRC tumor progression.

### 3.6. miR-429 Directly Targets LATS2 mRNA

**Figure 4:**
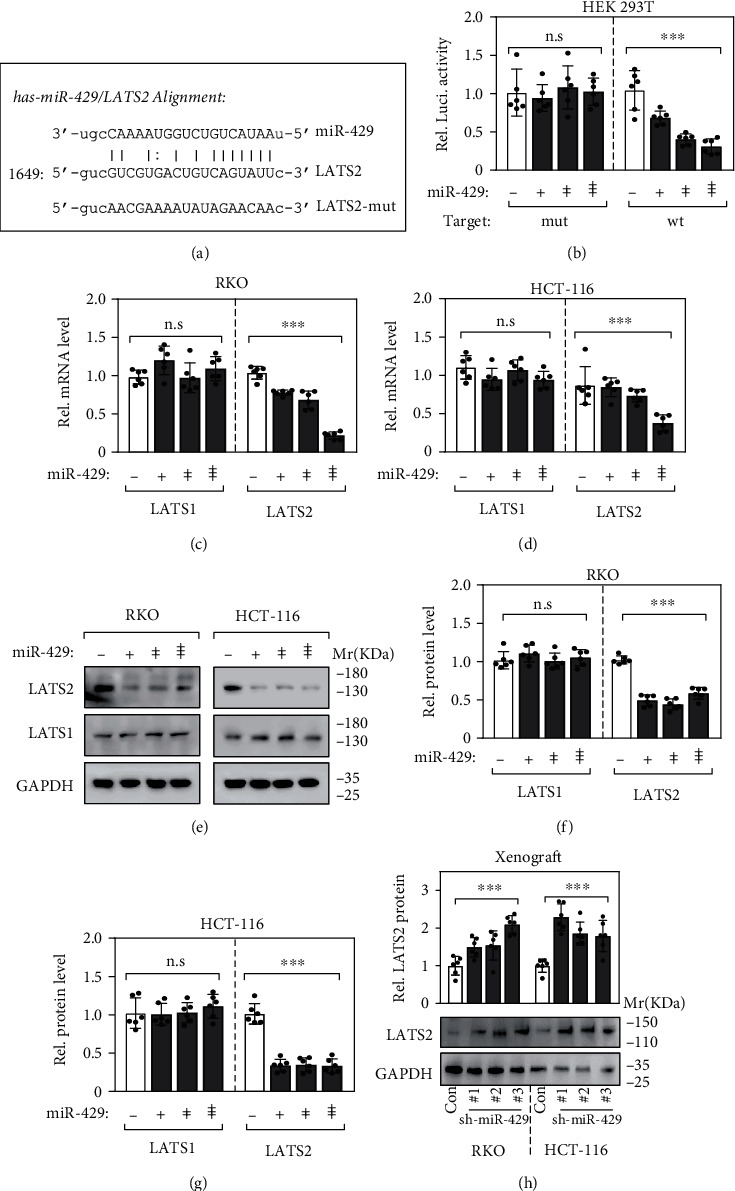
miR-429 directly targets LATS2 mRNA. (a) Alignment of has-miR-429 and the 3′-UTR sequences of LATS2 and the mutant miR429 binding sequence in the 3′-UTR of LATS2. (b) Dual-luciferase reporter assay showed the direct interaction between miR429 and LATS2. (c, d) The mRNA levels of LATS2 but not LATS1 in RKO and HCT116 cells reduced by overexpression of miR-429. (e–g) Western blotting analysis confirmed that LATS2 but not LATS1 was downregulated by overexpression of miR-429. (h) Western blotting analysis confirmed that LATS2 protein expression levels were retrieved in the xenografts of sh-miR-429.

microRNAs regulate mRNAs expression by targeting the 3′-UTR of the mRNAs with complementary sequences. Using the TargetScan program, we found that there were complementary binding sites of miR-429 in the 3′-UTR sequences of LATS2 (Figure 4(a)), suggesting that miR-429 was virtually a potential regulator of LATS2.

To verify their interaction, luciferase reporters and a dual-luciferase reporter assay system were applied according to the manufacturer. Briefly, the wild-type (WT) or the mutant miR429 binding sequences in the 3′-UTR of LATS2 were cloned into the downstream of the firefly luciferase reporter gene, then cotransfected with miR-429 mimic or an unrelated sequence as a control into HEK 293T cells. As shown in Figure 4(b), when miR-429 was overexpressed, the relative luciferase activities were significantly suppressed in cells carrying the WT 3′-UTR of LATS2 (Figure 4(b)).

To verify the direct regulation of miR-429 on LATS2 expression, we transfected RKO and HCT116 cell lines with miR-429 mimics and detected the expression of LATS2. Both quantitative real-time PCR and western blotting analyses revealed that the LATS2 expression levels but not LATS1 were reduced in miR-429-expressing cells (Figures 4(c)–4(g)), indicating that miR-429 directly bound to LATS2 at the predicted binding site in its 3′-UTR sequence and negatively regulated its expression. Furthermore, shRNA interfering actually retrieved the protein expression levels of LATS2 in the tumor xenografts (Figure 4(h)).

### 3.7. miR-429 Promotes Tumorigenesis in a LATS2-Dependent Manner

**Figure 5:**
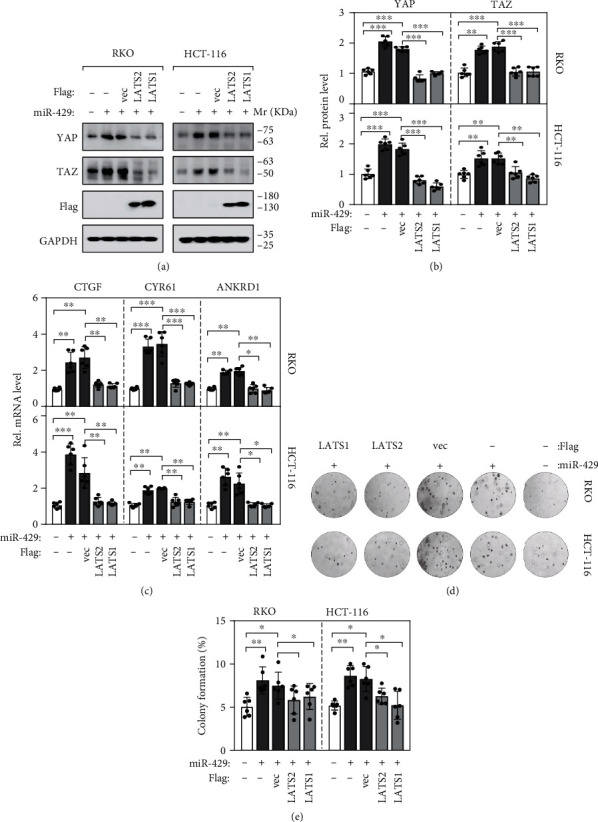
miR-429 promotes tumorigenesis in a LATS2 dependent manner. (a, b) Western blotting analysis of YAP/TAZ expressions in miR-429 mimics transfected RKO and HCT116 cells with or without LATS1/2 flag. (c) Expressions of the downstream molecules of YAP/TAZ, CTGF, CYR61, and ANKRD1 were affected by miR-429 and LAST1/2 accordingly. (d, e) The enhanced colony formation abilities of RKO and HCT116 cells by forced expression of miR-429 were retrieved by LAST1/2 overexpression.

To further validate that LATS2 was a direct target of miR-429 and determine whether miR-429 suppresses cancer cell proliferation and growth by regulating LATS2 and then YAP/TAZ expression, flag-tagged LATS1 and LATS2 overexpressing plasmids were constructed and transfected into RKO or HCT116 cell line together with miR-429 mimics. Overexpression of LATS1/2 was confirmed by western blotting analysis (Figure 5(a)), and the effects on YAP/TAZ expression level and their downstream target molecules were evaluated. As shown in Figures 5(a) and 5(b), forced expression of miR-429 significantly increased the expression levels of YAP/TAZ, while cotransfection of LATS2 or LATS1 together with miR-429 mimics significantly decreased their expressions. Apart from this, the expression levels of the YAP/TAZ target molecules such as CTGF, CYR61, and ANKRD1 have been also influenced accordingly (Figure 5(c)).

Cellular experiments of colony formation assays showed that the restoration of LATS1/2 expression abrogated the tumor-promoting effects of miR-429 as that miR-429-mediated promotion of cell proliferation was markedly suppressed by cooverexpression of LATS1/2 in RKO and HCT116 cells compared to those with the vector controls (Figures 5(d) and 5(f)).

## 4. Discussion

In this study, we first detected expressions of YAP/TAZ in 21 paired COAD and the adjacent noncancer tissue samples and found that the YAP/TAZ expression levels were greatly increased in the tumor tissues at the protein levels but not at the mRNA levels. As the key serine/threonine kinase and the main regulator of YAP/TAZ in the Hippo signaling pathway, downregulation of LATS2, but not LATS1, contributed to YAP/TAZ activation. Furthermore, we identified miR-429 as the involved miRNA regulator in LATS2-YAP/TAZ signaling. Functional assays showed that tumor growth and colony-formation rates were markedly slower in miR-429-silenced CRC cells than in the corresponding control cells. We proposed that the increased expressions of YAP/TAZ and their oncogenic function in colon cancer might due to the downregulation of LATS2 kinase by overexpressing of miR-429.

As the main effecter of the Hippo signaling and the hub of many pathways including Hippo, Notch, Wnt/*β*-catenin, and TGF-*β*/Smad signaling, YAP/TAZ dysregulation contributes to the genesis and development of many kinds of cancers. LATS1/2, as the main regulators, are supposed to phosphorylate YAP/TAZ and eventually lead to their degradation [23]. LATS2 has been mostly proved to be downregulated in human cancers including colorectal cancer, breast cancer, and hepatocellular carcinoma. It is reported that LATS2 suppression promoted EMT and cancer metastasis through enhancing mitochondrial ROS production in liver cancer [25]. And overexpression of LATS2 in breast cancer suppressed cell invasion through controlling tumor glucose metabolism [26].

miRNAs are short noncoding RNA molecules of approximately 19-24 nucleotides, involved in many processes of tumor growth such as proliferation, apoptosis, migration, invasion, and drug resistance. Expression of LATS2 could be affected by kinds of microRNAs such as miR-93 and miR-372. Guo and his colleagues reported that the occurrence of prostate cancer was related to the downregulation of LATS2 and the upregulation of YAP and miR-302/367 cluster. They found out that miR-302/367 downregulated LATS2 expression and reduced the phosphorylation of YAP, and then resulted in enhanced YAP nuclear translocation in prostate tumor-propagating cells [27]. Studies of Li et al. showed that the upregulation of miR-373 promoted the migration, invasion, and proliferation of endometrial cancer cells via downregulating LATS2 [28]. LATS2 was also reported to be regulated by many other microRNAs such as miR-93 [29], miR-372 [30], and miR-744 [31] in different cancers.

miR-429 is a miR-200 family member. It has been recently found to be differentially expressed in various cancers and might play important roles during carcinogenesis. As reported, the expression levels of miR-429 were significantly higher in renal cancer patients compared with those normal patients [32], and overexpression of miR-429 increased the cell proliferation rate and decreased the apoptosis rate significantly. Another two studies separately identified miR429 as a tumor promoter in ovarian cancer as that both comprehensive bioinformational analysis and serum expression detection experiment showed overexpressing of miR-429 [33, 34]. Nevertheless, numerous findings were suggesting that miR-429 might act as a tumor suppressor as well on the development and progression of several cancers. It was shown to be downregulated and might function as a tumor suppressor by targeting Myc [35], FSCN1 [36] in gastric cancer, ZEB1 [37] in oral squamous cell carcinoma, CRKL [38] in hepatocellular carcinoma, TLN1 [39] in nasopharyngeal carcinoma, AKT1 in melanoma [40], or BCL2 and SP1 in esophageal carcinomas [41].

In colon cancer, there were several studies showed that miR429 inhibited colon cancer cell growth. Sun and coworkers found that it was significantly downregulated in stage II and III clinical progressions of colorectal cancer [42]. miR-429 was able to inhibit the invasion of carcinoma to the other organs [43]. On the contrary, Li and coworkers showed that miR-429 was upregulated in human CRC tissues and that overexpression of miR-429 inhibited CRC cell apoptosis through negatively regulate SOX2 expression [44]. Functional studies by Han and colleagues also suggested that miR-429 played an oncogenic role in the cellular procession of CRC [45]. Supporting this, our present study revealed that miR-429 could upregulate YAP/TAZ expression and promote COAD growth in CRC by directly targeting LATS2, suggesting that miR-429 played an oncogenic role in CRC.

The mRNA expression levels in the abovementioned literature were based on RNA extraction. It is conceivable that the contribution of nontumor cells, the variable volume of stroma in the tissues, could be confounding factors in the expression level determination when using whole tissue for RNA extraction. When using laser capture microdissection (LCM) as part of the methodologies, Bojmar et al. found that miR-141 was significantly decreased in the primary tumor versus controls, while there was no difference in its expression between the groups when total tumor tissue was used for RNA extraction [46]. The study of Paterson et al. also used LCM for tissue dissection and found the different expression patterns of miR-200b at the invasive front of tumors compared to normal and tumor core tissues [47]. Biogenesis and expression of miRNAs are subjected to strict temporal and spatial control, and the different expression patterns of miRNAs might be associated with many clinical variables such as histological type, differentiation degree, tumor location, and tumor grading and staging in these cancers. These variables might also account for some of the aberrations in the above study results. Investigators should carefully consider these important factors when deciphering the findings and making conclusions.

## 5. Conclusion

Collectively, we discovered a new signaling axis of the Hippo pathway, miR-429-LATS2-YAP1/TAZ, in colon cancer through bioinformatical gene expression screening and functional analysis, offering a plausible mechanism accounting for the tumor-promoting function of YAP/TAZ. We suppose that targeting this signaling axis might represent a promising therapeutic strategy for colorectal cancer.

## Data Availability

The data used to support the findings of this study are available from the corresponding author upon request.

## References

[1] Siegel R. L., Miller K. D., Fedewa S. A. (2017). Colorectal cancer statistics, 2017. *CA: a Cancer Journal for Clinicians*.

[2] Buskermolen M., Cenin D. R., Helsingen L. M. (2019). Colorectal cancer screening with faecal immunochemical testing, sigmoidoscopy or colonoscopy: a microsimulation modelling study. *BMJ*.

[3] Helsingen L. M., Vandvik P. O., Jodal H. C. (2019). Colorectal cancer screening with faecal immunochemical testing, sigmoidoscopy or colonoscopy: a clinical practice guideline. *BMJ*.

[4] Li M., Jiang X., Wang G. (2019). ITGB4 is a novel prognostic factor in colon cancer. *Journal of Cancer*.

[5] Tao Y., Cai F., Shan L., Jiang H., Ma L., Yu Y. (2017). The Hippo signaling pathway: an emerging anti-cancer drug target. *Discovery Medicine*.

[6] Pfleger C. M. (2017). The Hippo pathway: a master regulatory network important in development and dysregulated in disease. *Current Topics in Developmental Biology*.

[7] Zanconato F., Cordenonsi M., Piccolo S. (2016). YAP/TAZ at the roots of cancer. *Cancer Cell*.

[8] Zhang H., Geng D., Gao J. (2016). Expression and significance of Hippo/YAP signaling in glioma progression. *Tumor Biology*.

[9] Liu M., Lin Y., Zhang X. C. (2017). Phosphorylated mTOR and YAP serve as prognostic markers and therapeutic targets in gliomas. *Laboratory Investigation*.

[10] Dong L., Lin F., Wu W., Huang W., Cai Z. (2016). Transcriptional cofactor Mask2 is required for YAP-induced cell growth and migration in bladder cancer cell. *Journal of Cancer*.

[11] Kim T., Yang S. J., Hwang D. (2015). A basal-like breast cancer-specific role for SRF-IL6 in YAP-induced cancer stemness. *Nature Communications*.

[12] Basu-Roy U., Bayin N. S., Rattanakorn K. (2015). Sox2 antagonizes the Hippo pathway to maintain stemness in cancer cells. *Nature Communications*.

[13] Cordenonsi M., Zanconato F., Azzolin L. (2011). The Hippo transducer TAZ confers cancer stem cell-related traits on breast cancer cells. *Cell*.

[14] Wang L., Shi S., Guo Z. (2013). Overexpression of YAP and TAZ is an independent predictor of prognosis in colorectal cancer and related to the proliferation and metastasis of colon cancer cells. *PLoS One*.

[15] Yuan L., Zhou M., Wasan H. S. (2019). Jiedu Sangen decoction inhibits the invasion and metastasis of colorectal cancer cells by regulating EMT through the Hippo signaling pathway. *Evidence-based Complementary and Alternative Medicine*.

[16] Zhang Y., Fang Z., Guo X. (2019). lncRNA B4GALT1-AS1 promotes colon cancer cell stemness and migration by recruiting YAP to the nucleus and enhancing YAP transcriptional activity. *Journal of Cellular Physiology*.

[17] Chai Y., Xiang K., Wu Y. (2018). Cucurbitacin B Inhibits the hippo-YAP Signaling pathway and exerts anticancer activity in colorectal Cancer cells. *Medical Science Monitor*.

[18] Zhou B., Zong S., Zhong W. (2020). Interaction between laminin-5*γ*2 and integrin *β*1 promotes the tumor budding of colorectal cancer via the activation of Yes-associated proteins. *Oncogene*.

[19] Greenhough A., Bagley C., Heesom K. J. (2018). Cancer cell adaptation to hypoxia involves a HIF-GPRC5A-YAP axis. *EMBO Molecular Medicine*.

[20] Lu J., Getz G., Miska E. A. (2005). MicroRNA expression profiles classify human cancers. *Nature*.

[21] Ambros V. (2004). The functions of animal microRNAs. *Nature*.

[22] Kim C. L., Choi S. H., Mo J. S. (2019). Role of the hippo pathway in fibrosis and cancer. *Cell*.

[23] Mo J. S., Park H. W., Guan K. L. (2014). The Hippo signaling pathway in stem cell biology and cancer. *EMBO Reports*.

[24] Moroishi T., Hansen C. G., Guan K. L. (2015). The emerging roles of YAP and TAZ in cancer. *Nature Reviews. Cancer*.

[25] Han L. L., Yin X. R., Zhang S. Q. (2018). miR-650 promotes the metastasis and epithelial-mesenchymal transition of hepatocellular carcinoma by directly inhibiting LATS2 expression. *Cellular Physiology and Biochemistry*.

[26] Furth N., Pateras I. S., Rotkopf R. (2018). LATS1 and LATS2 suppress breast cancer progression by maintaining cell identity and metabolic state. *Life Sci Alliance*.

[27] Guo Y., Cui J., Ji Z. (2017). miR-302/367/LATS2/YAP pathway is essential for prostate tumor-propagating cells and promotes the development of castration resistance. *Oncogene*.

[28] Li Y., Sun D., Gao J. (2019). MicroRNA-373 promotes the development of endometrial cancer by targeting LATS2 and activating the Wnt/beta-Catenin pathway. *Journal of Cellular Biochemistry*.

[29] Liu J. J., Zhang X., Wu X. H. (2018). miR-93 promotes the growth and invasion of prostate cancer by upregulating its target genes TGFBR2, ITGB8, and LATS2. *Molecular Therapy - Oncolytics*.

[30] Cheng X., Chen J., Huang Z. (2018). miR-372 promotes breast cancer cell proliferation by directly targeting LATS2. *Experimental and Therapeutic Medicine*.

[31] Sun L., Liu M., Luan S., Shi Y., Wang Q. (2019). MicroRNA-744 promotes carcinogenesis in osteosarcoma through targeting LATS2. *Oncology Letters*.

[32] Wang Z. K., Luo L., Du Z. J., Zhang G. M., Sun L. J. (2017). MiR429 expression level in renal cell cancer and its correlation with the prognosis of patients. *Journal of BUON*.

[33] Wu L., Shang W., Zhao H. (2019). In silico screening of circulating microRNAs as potential biomarkers for the diagnosis of ovarian cancer. *Disease Markers*.

[34] Marton E., Lukacs J., Penyige A. (2019). Circulating epithelial-mesenchymal transition-associated miRNAs are promising biomarkers in ovarian cancer. *Journal of Biotechnology*.

[35] Sun T., Wang C., Xing J., Wu D. (2011). miR-429 modulates the expression of c-myc in human gastric carcinoma cells. *European Journal of Cancer*.

[36] Zhang M., Dong B. B., Lu M. (2016). miR-429 functions as a tumor suppressor by targeting FSCN1 in gastric cancer cells. *Oncotargets and Therapy*.

[37] Lei W., Liu Y. E., Zheng Y., Qu L. (2015). MiR-429 inhibits oral squamous cell carcinoma growth by targeting ZEB1. *Medical Science Monitor*.

[38] Guo C., Zhao D., Zhang Q., Liu S., Sun M. Z. (2018). miR-429 suppresses tumor migration and invasion by targeting CRKL in hepatocellular carcinoma *via* inhibiting Raf/MEK/ERK pathway and epithelial- mesenchymal transition. *Scientific Reports*.

[39] Wang Z., Zhu Z., Lin Z. (2019). miR-429 suppresses cell proliferation, migration and invasion in nasopharyngeal carcinoma by downregulation of TLN1. *Cancer Cell International*.

[40] Huang D., Wang F., Wu W., Lian C., Liu E. (2019). MicroRNA-429 inhibits cancer cell proliferation and migration by targeting the AKT1 in melanoma. *Cancer Biomarkers*.

[41] Wang Y., Li M., Zang W. (2013). MiR-429 up-regulation induces apoptosis and suppresses invasion by targeting Bcl-2 and SP-1 in esophageal carcinoma. *Cellular Oncology*.

[42] Sun Y., Shen S., Tang H. (2014). miR-429 identified by dynamic transcriptome analysis is a new candidate biomarker for colorectal cancer prognosis. *OMICS*.

[43] Sun Y., Shen S., Liu X. (2014). MiR-429 inhibits cells growth and invasion and regulates EMT-related marker genes by targeting Onecut2 in colorectal carcinoma. *Molecular and Cellular Biochemistry*.

[44] Li J., Du L., Yang Y. (2013). MiR-429 is an independent prognostic factor in colorectal cancer and exerts its anti-apoptotic function by targeting SOX2. *Cancer Letters*.

[45] Han Y., Zhao Q., Zhou J., Shi R. (2017). miR-429 mediates tumor growth and metastasis in colorectal cancer. *American Journal of Cancer Research*.

[46] Bojmar L., Karlsson E., Ellegård S. (2013). The role of microRNA-200 in progression of human colorectal and breast cancer. *PLoS One*.

[47] Emily L., Paterson J. K., Bert A. G., Khew-Goodall Y., Ruszkiewicz A., Goodall G. J. (2013). Down-regulation of the miRNA-200 family at the invasive front of colorectal cancers with degraded basement membrane indicates EMT is involved in cancer progression. *Neoplasia*.

